# Correction: Mechanism unravelling for highly efficient and selective ^99^TcO_4_^−^ sequestration utilising crown ether based solvent system from nuclear liquid waste: experimental and computational investigations

**DOI:** 10.1039/d2ra90015g

**Published:** 2022-02-18

**Authors:** Kankan Patra, Arijit Sengupta, Anil Boda, Musharaf Ali, V. K. Mittal, T. P. Valsala, C. P. Kaushik

**Affiliations:** Nuclear Recycle Board, Bhabha Atomic Research Centre Tarapur 401504 India kankan.patra2010@gmail.com; Radiochemistry Division, Bhabha Atomic Research Centre Mumbai 400 085 India arijitbarc@gmail.com; Homi Bhabha National Institute Anushaktinagar Mumbai 400 094 India; Chemical Engineering Division, Bhabha Atomic Research Centre Mumbai 400 085 India; Nuclear Recycle Group, Bhabha Atomic Research Centre Mumbai 400 085 India

## Abstract

Correction for ‘Mechanism unravelling for highly efficient and selective ^99^TcO_4_^−^ sequestration utilising crown ether based solvent system from nuclear liquid waste: experimental and computational investigations’ by Kankan Patra *et al.*, *RSC Adv.*, 2022, **12**, 3216–3226. DOI: 10.1039/D1RA07738D.

In the original manuscript in Fig. 8, part of the cover image from *Analytical Sciences*, 2020, **36** (12), 1433–1576 was used. This Correction article provides an acknowledgement of the source of that part of the image for Fig. 8 after permission was granted by the corresponding author for the *Analytical Sciences* paper.

## Supplementary Material

